# Genome-wide characterization and expression analysis of the HD-Zip gene family in response to drought and salinity stresses in sesame

**DOI:** 10.1186/s12864-019-6091-5

**Published:** 2019-10-16

**Authors:** Mengyuan Wei, Aili Liu, Yujuan Zhang, Yong Zhou, Donghua Li, Komivi Dossa, Rong Zhou, Xiurong Zhang, Jun You

**Affiliations:** 1 Key Laboratory of Biology and Genetic Improvement of Oil Crops, Ministry of Agriculture and Rural Affairs, Oil Crops Research Institute of the Chinese Academy of Agricultural Sciences, Wuhan, 430062 China; 2 Special Economic Crop Research Center of Shandon Academy of Agricultural Sciences, Shandong Cotton Research Center, Jinan, 250100 China; 3 Key Laboratory of Crop Physiology, Ecology and Genetic Breeding, Ministry of Education, Jiangxi Agricultural University, Nanchang, 330045 China

**Keywords:** Sesame, Transcription factors, HD-ZIP, Abiotic stress, Gene expression

## Abstract

**Background:**

The homeodomain-leucine zipper (HD-Zip) gene family is one of the plant-specific transcription factor families, involved in plant development, growth, and in the response to diverse stresses. However, comprehensive analysis of the HD-Zip genes, especially those involved in response to drought and salinity stresses is lacking in sesame (*Sesamum indicum* L.), an important oil crop in tropical and subtropical areas.

**Results:**

In this study, 45 HD-Zip genes were identified in sesame, and denominated as SiHDZ01-SiHDZ45. Members of SiHDZ family were classified into four groups (HD-Zip I-IV) based on the phylogenetic relationship of *Arabidopsis* HD-Zip proteins, which was further supported by the analysis of their conserved motifs and gene structures. Expression analyses of *SiHDZ* genes based on transcriptome data showed that the expression patterns of these genes were varied in different tissues. Additionally, we showed that at least 75% of the *SiHDZ* genes were differentially expressed in responses to drought and salinity treatments, and highlighted the important role of HD-Zip I and II genes in stress responses in sesame.

**Conclusions:**

This study provides important information for functional characterization of stress-responsive HD-Zip genes and may contribute to the better understanding of the molecular basis of stress tolerance in sesame.

## Background

Transcription factors (TFs) play a vital role in regulatory networks that link between the developmental program and response of genes to endogenous and environmental signals. TFs directly bind to the promoters of target genes in a sequence-specific manner to activate or repress the expression of their target genes [1]. Homeobox proteins are a large superfamily of transcription factors (TFs) found in invertebrates, vertebrates, fungi, and plants, which contain a highly conserved DNA-binding domain known as the homeodomain (HD) [2]. The HD is 60 amino acids in length and adopts a structure of three α-helices connected by a loop and a turn [2–4]. The HD binds DNA as a monomer with high affinity through the interactions established by helix III (called the recognition helix) with the major groove of the target DNA while the disordered N-terminal arm, located upstream the first helix, interacts with the DNA minor groove [5]. Homeodomain proteins have been classified into different families according to the distinguishing features, such as HD location, association with other domains, their size and gene structures. Based on thorough analyses of homeodomain proteins from flowering plants, moss, *Selaginella*, unicellular green algae, and red algae, Mukherjee et al. [6] classified the plant HD-containing proteins into 14 families, including HD-Zip (homeodomain associated to a leucine zipper), WOX (Wuschel related homeobox) and KNOX (Knotted related homeobox).

The plant-specific HD-Zip transcription factors contain a leucine zipper motif (LZ) that acts as a dimerization motif, located in the carboxyl terminal to the homeodomain [2, 6]. The HD-ZIP family further divided into four subfamilies, HD-Zip I-IV, according to the conserved HD-Zip domain, additional conserved domain, structure features and functions [6]. Members of HD-Zip I and II family share the conserved HD and LZ domains, and bind similar pseudo-palindromic cis-elements, CAATNATTG, where N can be A/T or G/C for HD-Zip I or II homeodomains, respectively [7, 8]. Besides HD and LZ domains, HD-ZIP II proteins present a conserved CPSCE motif (named by five conserved amino acids: Cys, Pro, Ser, Cys and Glu) downstream of the LZ [9]. Both the HD-Zip III and HD-Zip IV subfamily proteins are characterized by the START (steroidogenic acute regulatory protein–related lipid transfer) domain and START adjacent domain (SAD) [6, 10]. HD-Zip III proteins can be distinguished from HD-Zip IV proteins by the presence of C-terminal MEKHLA domain which is absent in the HD-ZIP IV proteins [11]. HD-Zip III proteins bind the consensus sequence GTAAT [G/C] ATTAC, while members of HD-Zip IV family recognized the sequence TAAATG [C/T] A [12, 13].

There are 48 and 49 HD-ZIP genes in *Arabidopsis thaliana* and rice (*Oryza sativa* L.), respectively [6, 14, 15]. Through genome-wide analysis, members of HD-Zip gene family have been also identified in many other plant species, including grape (*Vitis vinifera*) [16], maize (*Zea mays*) [17], soybean (*Glycine max*) [18, 19], cassava (*Manihot esculenta*) [20], wheat (*Triticum aestivum*) [21], tea plant (*Camellia sinensis*) [22], and potato (*Solanum tuberosum*) [23]. HD-Zip proteins are known to participate in transcriptional regulation of various biological processes, and members of the different subfamilies have specific roles [2, 24, 25]. HD-Zip I proteins were found to be implicated in the regulation of abiotic stress responses, light and hormone (ABA, auxin and ethylene) signal transduction, and plant growth and development [2, 24, 26]. *AtHB7* (*Arabidopsis thaliana HOMEOBOX 7*) and *AtHB12* (*Arabidopsis thaliana HOMEOBOX 12*) from *Arabidopsis* HD-Zip I group participate in ABA sensing and transduction, playing a key role in drought and salt responses [27, 28]. MtHB1 (*MEDICAGO TRUNCATULA* HOMEOBOX 1), a HD-Zip I protein from *M. truncatula*, regulates root architecture under adverse environmental stresses by repressing *LBD1* (*LOB-BINDING DOMAIN 1*) involving crosstalk between auxin and ABA signaling pathways [29]. In the case of HD-Zip II proteins, they are mainly involved in development, shade avoidance and abiotic stress responses [30–32]. For example, members of the HD-Zip II family, including AtHB2 (*Arabidopsis thaliana* HOMEOBOX 2), AtHB4 (*Arabidopsis thaliana* HOMEOBOX 4), and HAT3 (*HOMEOBOX FROM Arabidopsis thaliana 3*), play crucial roles in regulation of leaf polarity and shade avoidance response [31, 33]. Two other HD-Zip II proteins, AtHB17 (*Arabidopsis thaliana* HOMEOBOX 17) and ABIG1 (ABA INSENSITIVE GROWTH 1), are involved in ABA-mediated stress response or growth inhibition [34, 35]. HD-Zip III proteins were reported to be involved in apical meristem formation, vascular development, organ polarity establishment, as well as auxin biosynthesis, transport and response [31, 36]. Rice *LF1* (*LATERAL FLORET 1*) gene, encoding a class III HD-ZIP protein, induced the three-florets spikelet by directly regulating the expression of meristem maintenance gene *OSH1* (*ORYZA sativa HOMEOBOX 1*) [37]. HD-Zip IV proteins play critical role in the specification of the protoderm, anthocyanin accumulation, and environmental responses [38]. For example, GhHOX3 (*GOSSYPIUM HIRSUTUM* HOMEOBOX 3) in this subfamily plays a central role in controlling cotton fibre elongation [39].

Sesame is an ancient and important oil crop, which is grown mainly in tropical and subtropical areas of the world. Sesame has been widely used in baked and confectionery products and edible oil due to its highly stable oil and high quantities of nutritious amino acids, minerals, vitamins, and lignans [40]. However, sesame production and quality is threatened by drought, salinity and other environmental stresses [41–43]. A series of TFs, such as ERF, WRKY, MYB, NAC and bZIP, have been genome-wide analyzed in sesame, and some stress-responsive TFs have been identified [44–48]. However, response to abiotic stress of HD-Zip genes was unclear in sesame. In this study, we systematically characterized the HD-Zip gene family in sesame, and analyzed their phylogenetic relationships, conserved motifs and gene structure, as well as expression patterns in different tissues and in response to abiotic stresses. Our results provide a perspective for further investigation of the functions of stress-responsive HD-ZIPs in sesame.

## Results

### Genome-wide identification of HD-zip family genes in sesame

For the genome-wide identification of HD-Zip (homeodomain-leucine zipper) family genes in sesame, the Hidden Markov Model (HMM) profile of the homeodomain (HD) (PF00046) and the leucine zipper (LZ) domain (PF02183) were employed as queries to search against the Sinbase database (http://ocri-genomics.org/Sinbase) using the program HMM3.0. In addition, the known HD-Zip protein sequences from *Arabidopsis* were obtained from the TAIR database based on a previous study [2], and these sequences were also used as queries for searches in the Sinbase database. After removing redundant sequences, the SMART database was used to examine the presence of the HD and LZ domains for each identified candidate. As a result, a total of 45 *HD-Zip* genes were identified in sesame, and they were designated as *SiHDZ1*-*SiHDZ45* according to their chromosomal locations on the sesame linkage groups (LGs). The identified sesame *SiHDZ* genes encoded proteins ranging from a minimum of 160 (SiHDZ37) to a maximum of 847 (SiHDZ22) amino acids in length. Detail information of SiHDZs such as gene locus ID, linkage group location, proteins length, and other corresponding information are shown in Additional file 2: Table S1.

### Chromosomal localization and gene duplication analysis of SiHDZ genes

**Fig. 1:**
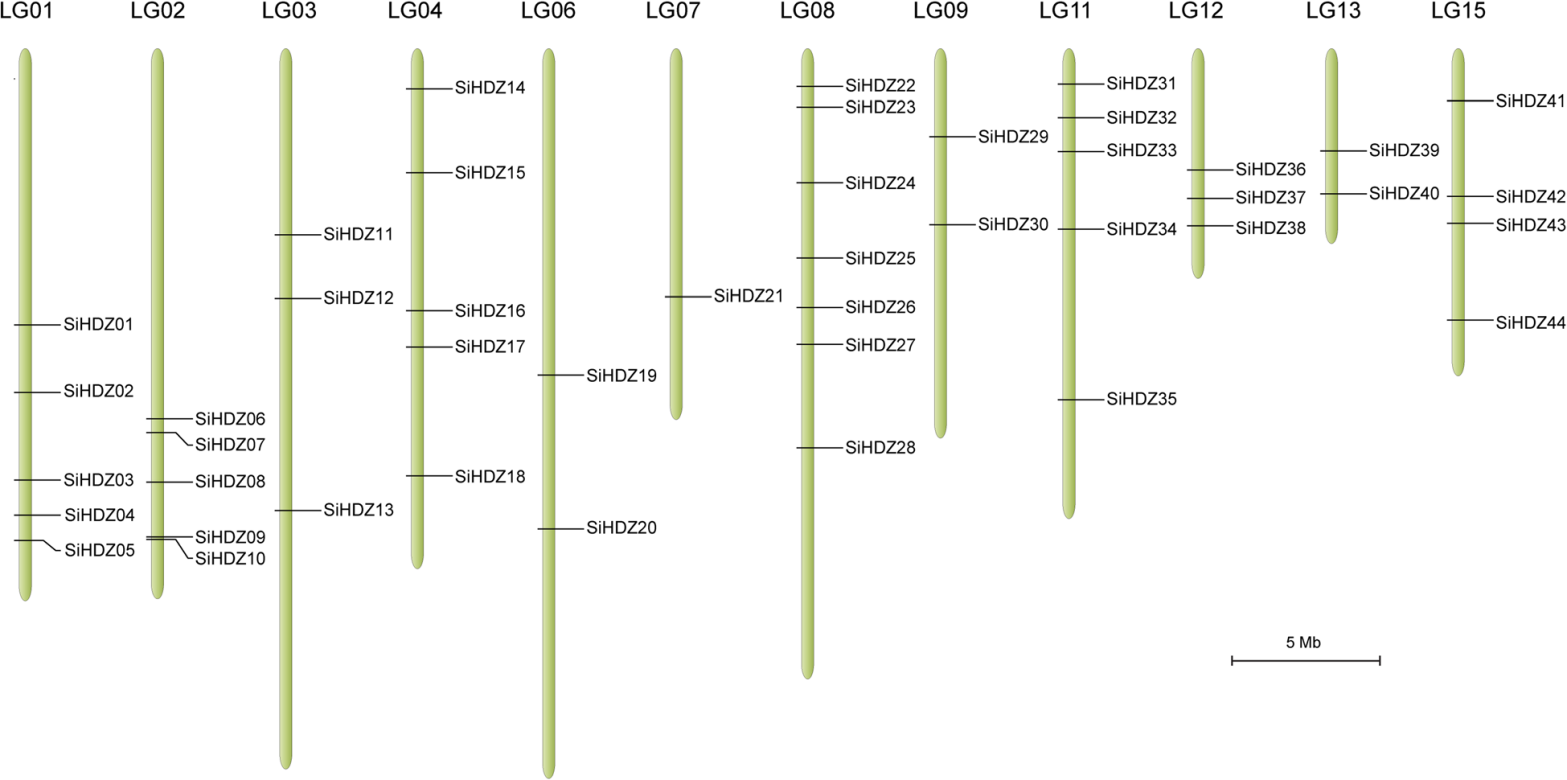
Distribution of *SiHDZ* genes on sesame linkage groups. The 44 *SiHDZ* genes were mapped onto 12 linkage groups (LGs) in the sesame genome. The LG number is indicated on the top of each LG. The scale bar represents 5 Mb

The chromosomal localization of *SiHDZ* genes was determined to visualize their genomic position information (Fig. 1). Of the 45 *SiHDZ* genes, 44 genes were distributed unequally on 12 out of the 16 LGs, with the LG08 having the majority of *SiHDZ* genes (7), whereas the LG07 had only one gene. In addition, one *SiHDZ* gene (*SiHDZ45*) was mapped to the unanchored scaffold, and is not shown in Fig. 1.

To study the potential gene duplication within the sesame genome, we examined the segmental and tandem duplication events in *SiHDZ* gene family during evolution. No tandem duplication events were found, while 23 gene pairs involving 36 *SiHDZ* genes resulted from segment duplication (Additional file 1: Figure S1).

### Phylogenetic analysis amongst the Arabidopsis and sesame HDZs

**Fig. 2:**
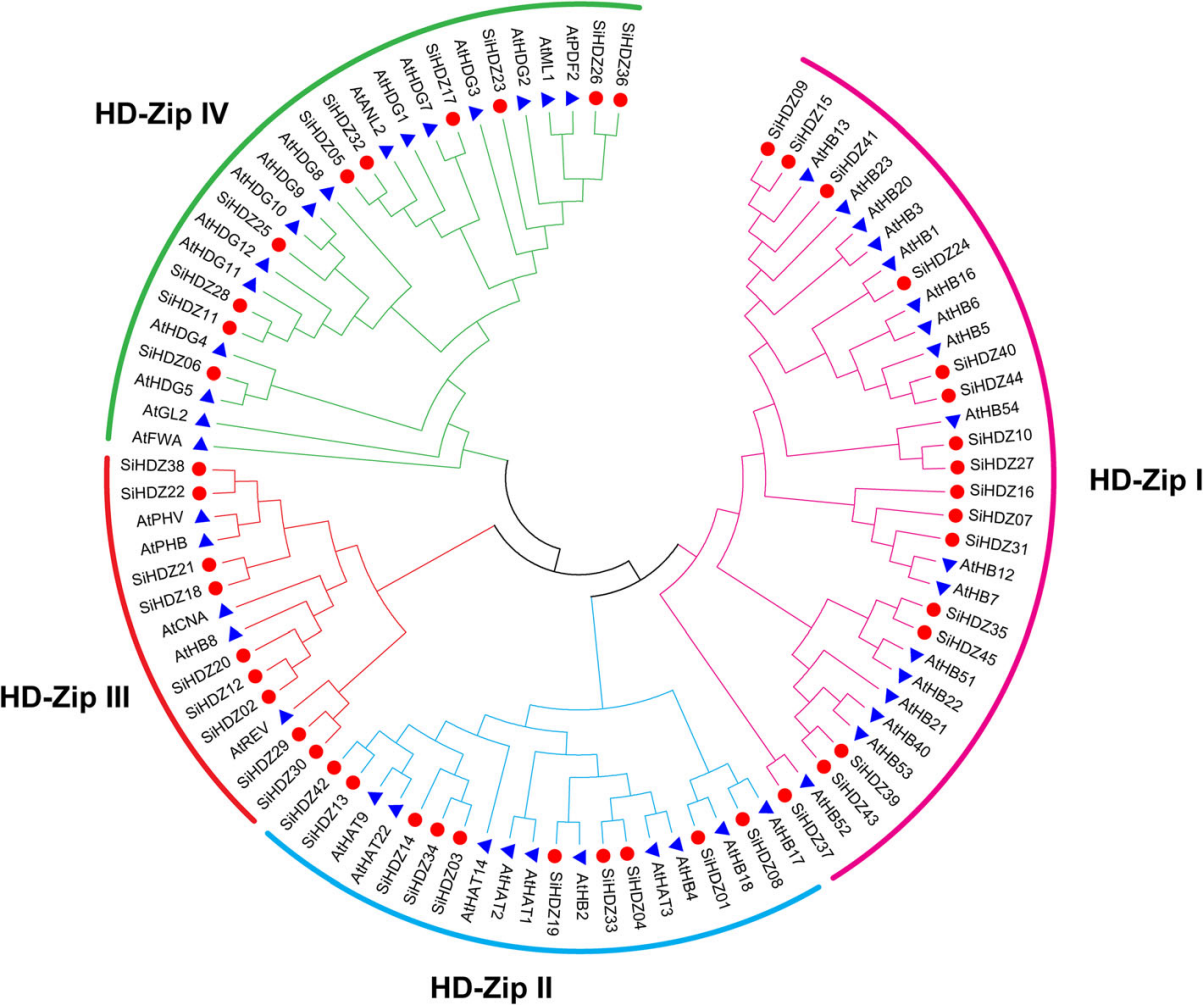
Phylogenetic analysis of HD-Zip proteins from sesame and *Arabidopsis*. The sequences were aligned by Clustal X and the tree was generated using the NJ method in MEGA 5.0 with 1000 bootstrap replicates. The blue triangles and red dots represent HD-Zip proteins in *Arabidopsis* and sesame, respectively

To reveal the phylogenetic relationships among the SiHDZ proteins, an unrooted phylogenetic tree was created to assess the genetic relationships between *Arabidopsis* and sesame HDZs. As shown in Fig. 2, these proteins can be divided into four distinct groups (HD-Zip I-IV), which is similar to that described in previous studies [2, 16]. The numbers of HD-Zip I-IV members in sesame were 16, 10, 9, and 10, respectively (Additional file 2: Table S1; Fig. 2). The results provide an important basis for functional prediction of HD-Zip proteins in sesame.

### Gene structure and conserved domain analysis of SiHDZ genes

**Fig. 3:**
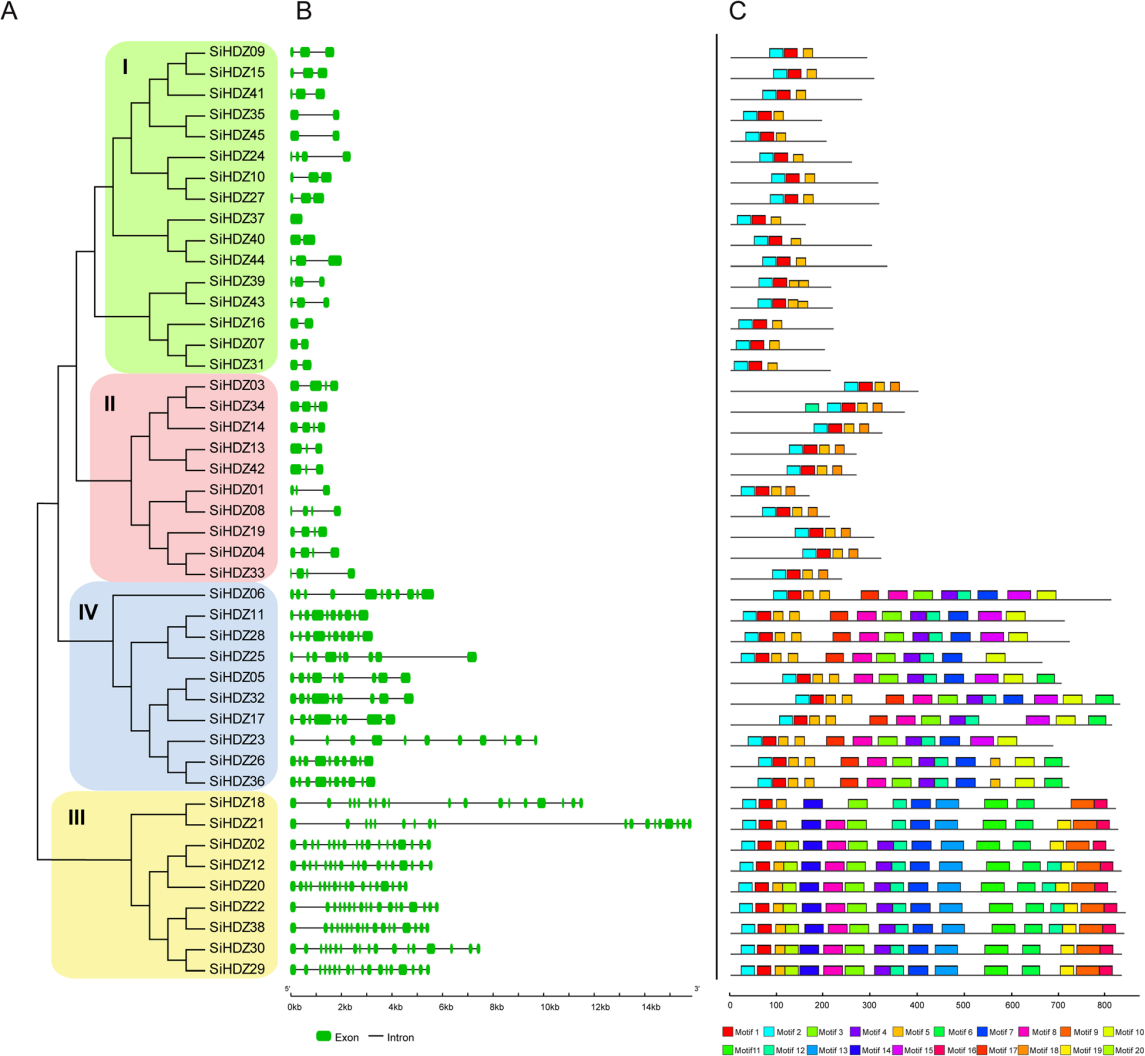
The exon-intron structure and conserved domain organization of *SiHDZ* genes. **a** Phylogenetic relationships of *SiHDZ* genes. The SiHDZ amino acid sequences were aligned by Clustal X and the tree was generated using the NJ method in MEGA 5.0 with 1000 bootstrap replicates. **b** exon–intron structures of *SiHDZ* genes. Exons and introns are shown as green boxes and black lines, respectively. **c** The conserved domain analysis of SiHDZ proteins using MEME. Different colors represent the different conserved motifs. The motif sequences are presented in Additional file 1: Figure S2

To investigate the structural diversity of *SiHDZ* genes, Gene Structure Display Server (http://gsds.cbi.pku.edu.cn/index.php) was employed to analyze the exon-intron structures of *SiHDZ* genes by comparing their coding sequences and corresponding genomic sequences. The results showed that the number of introns of *SiHDZ* genes varied between 0 and 17, but the genes that clustered together had similar number of introns (Fig. 3a, b). For example, all of the *SiHDZ* genes in group III contained the largest number of introns (17), members of *SiHDZ* group IV had 8–10 introns, with the exception of *SiHDZ17*, which contained 7 introns (Fig. 3b). Compared to group III and IV, *SiHDZ* genes in group I and II had much fewer introns. *SiHDZ* genes in group II usually contain 2–3 introns, whereas *SiHDZ* genes in group I feature 1–2 introns, except for *SiHDZ24* and *SiHDZ37*, whose number of introns were 3 and 0, respectively (Fig. 3b).

To further study the origin and evolutionary pattern of SiHDZs, their protein sequences were subjected to the MEME tool and a total of 20 conserved motifs were identified. The identified motifs ranged from 24 to 50 amino acids in length. The details of the sequence logo of each motif were presented in Additional file 1: Figure S2. Amongst them, motif 1 and 2, corresponding to the homeobox domain, and motif 5, corresponding to the LZ domain, were in common among all of the SiHDZs (Fig. 3c). In general, members in the same group harbored the similar motif organizations, while the motifs were divergent among different groups. For example, motifs 3, 4, 8, and 12, which correspond to START domain, were shared in group III and group IV, while motif 9 and 16, which correspond to MEKHLA domain, were present in group III but absent in group IV. In addition, motif 9 was exclusively present in group II, motif 13 and 19 only existed in group III, while motifs 10 and 17 was only present in the group IV HDZs (Fig. 3c). These group-specific motifs may imply diverse functions of the HDZ family in sesame.

### Expression profiles of SiHDZ genes in different tissues

**Fig. 4:**
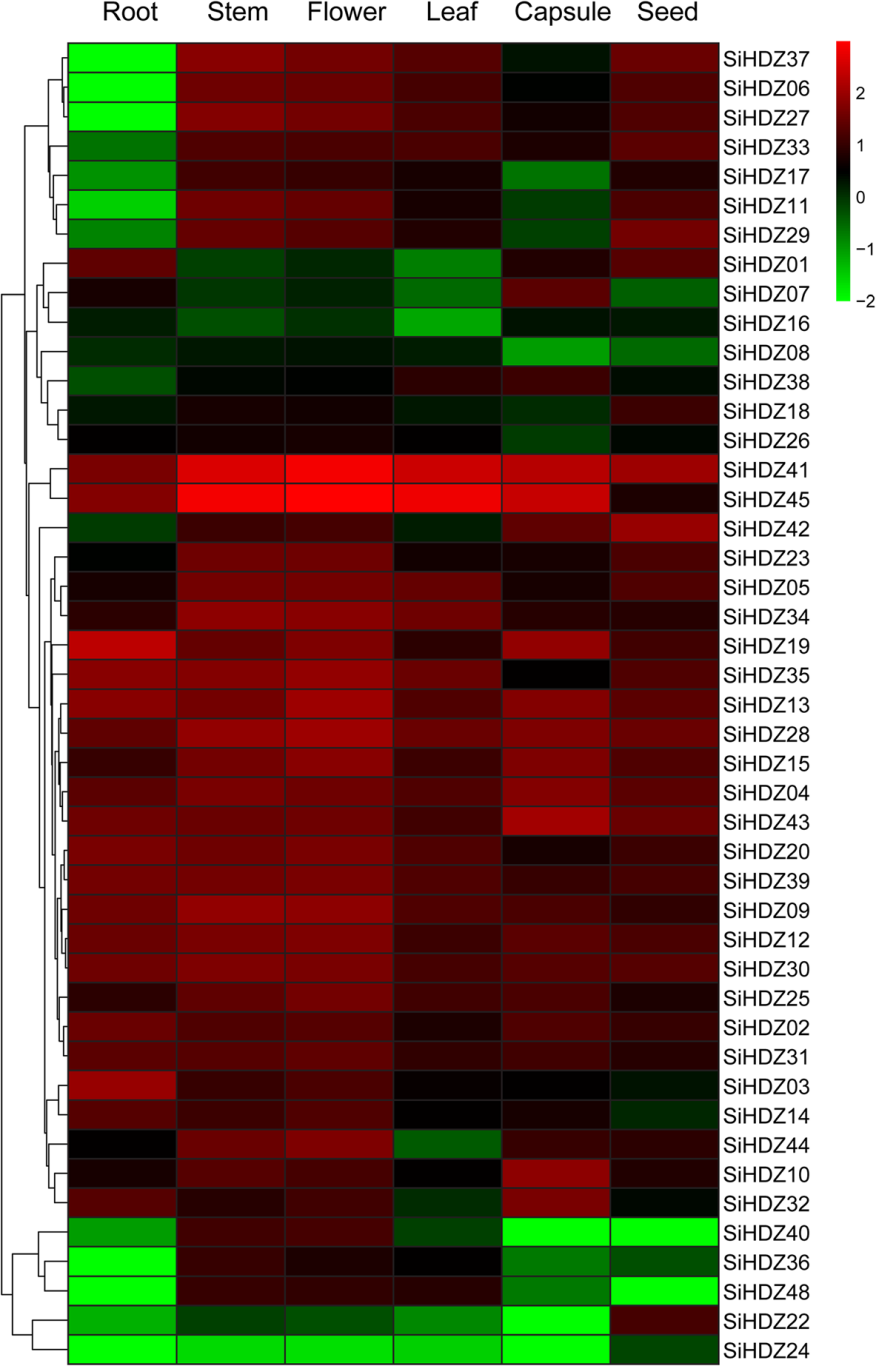
Cluster analysis of the *SiHDZs* expression profiles in root, stem, flower, leaf, capsule and seed. The heatmap shows the log10-transformed TPM values of each gene. The expression level is represented using color scale ranging from green (low expression) to red (high expression)

To study the potential functions of the *SiHDZ* genes, we analyzed the expression profiles of *SiHDZ* genes in different tissues, including root, stem, flower, leaf, capsule and seed, based on the transcriptome data from SesameFG database [49]. As shown in Fig. 4, 25 *SiHDZ* genes were commonly detected (TPM value > 1) in all of the tissues, suggesting that these genes might play a universal role in the tested tissues. Among them, four *SiHDZ* genes (*SiHDZ04*, *27*, *29*, and *40*) were highly expressed (TPM value > 10) in all six tissues, while *SiHDZ03* and *SiHDZ19* were most highly expressed (TPM value > 90) in root. In addition, some *SiHDZ* genes were shown to have tissue-specific expression (Fig. 4). For example, *SiHDZ11, 17, 28 and 38* displayed relatively low expression levels in root and capsule, but high expression in stem, flower, leaf and seed. *SiHDZ31* and *SiHDZ43* exhibited low expression levels in leaf, but relatively high expression levels in other tissues. The transcription levels of *SiHDZ01* and *SiHDZ07* were relatively higher in root and capsule, but low in stem, flower and leaf. Additionally, *SiHDZ21* showed specific expression in seed, whereas low in other tissues. These findings indicated the *SiHDZ* genes play differential roles in tissue development.

### Expression profiles of SiHDZ genes under drought stress

**Fig. 5:**
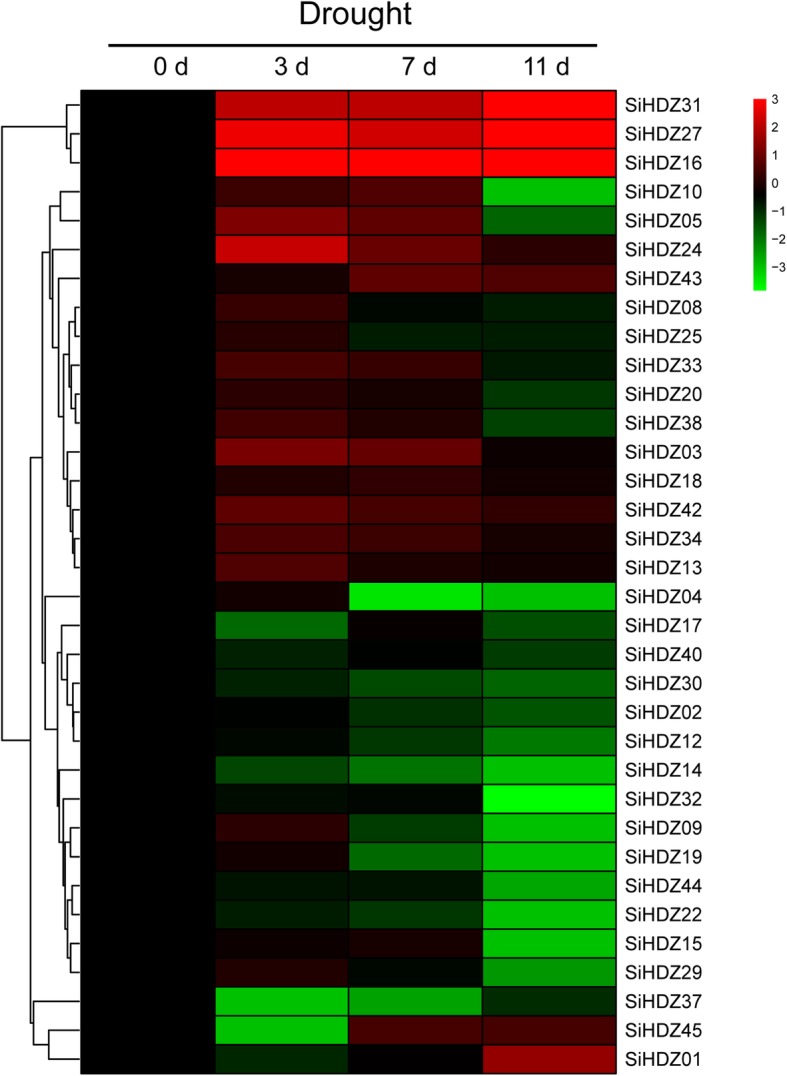
Cluster analysis of the *SiHDZs* expression profiles under drought stress**.** The heatmap was created based on the log2-transformed values of the relative expression levels of the *SiHDZ* genes under drought stress in drought-tolerant accession ZZM0635. The expression level is represented using color scale ranging from green (down-regulated) to red (up-regulated)

To identify the expression profiles of *SiHDZ* genes under drought stress, the expression pattern of each *SiHDZ* gene was obtained from the transcriptome data sets previously developed by our group [50]. It was observed that most of the *SiHDZ* genes were differentially affected under drought stress. For example, three *SiHDZ* genes, *SiHDZ16*, *SiHDZ27*, and *SiHDZ31*, were up-regulated under drought stress, with their expression markedly increasing at 3 d, and peaking at 11 d (Fig. 5). Some *SiHDZ* genes, such as *SiHDZ24*, *SiHDZ03*, and *SiHDZ42*, were also up-regulated under stress, but their expression levels were peaked at 3 d, implying their roles in early response to drought stress. However, some *SiHDZ* genes, such as *SiHDZ30*, *SiHDZ14*, *SiHDZ22*, and *SiHDZ37*, showed obvious down-regulation at all of the time points (Fig. 5). Moreover, some highly homologous SiHDZ genes exhibited similar expression profiles after drought stress, such as *SiHDZ16* and *SiHDZ31*, *SiHDZ19* and *SiHDZ04*, *SiHDZ12* and *SiHDZ02* (Fig. 5).

### Expression analysis of SiHDZ genes in response to osmotic and salinity stresses

**Fig. 6:**
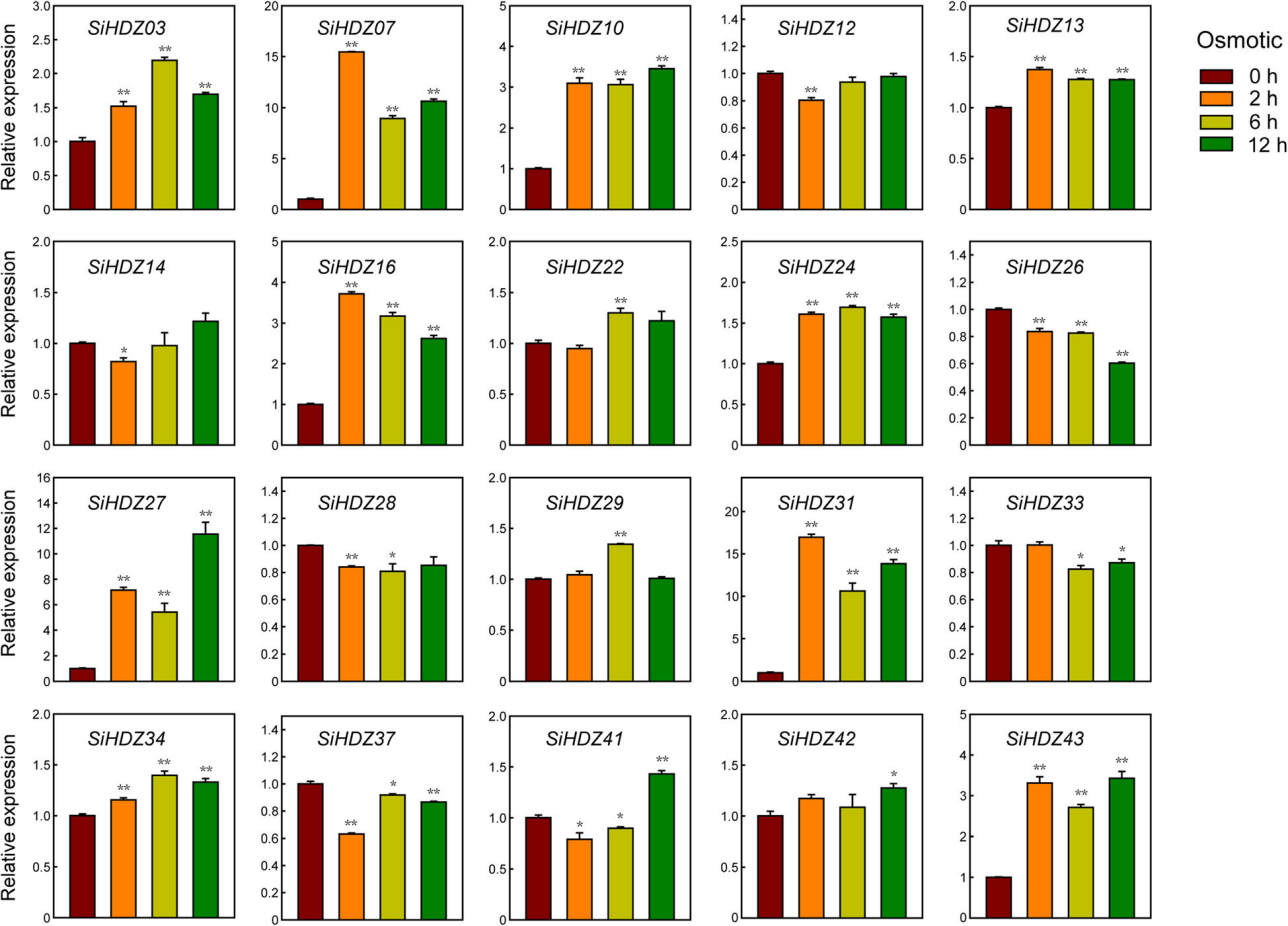
Expression patterns of 20 *SiHDZ* genes under osmotic stress. Two-week-old seedlings were subjected to osmotic (15% PEG 6000) stresses. Relative expression levels of *SiHDZ* genes were analyzed by qRT-PCR, using sesame *SiH3.3* gene as the internal control. Error bars indicate standard deviations (SD) based on three replicates. *P < 0.05; **P < 0.01, *t* test

**Fig. 7:**
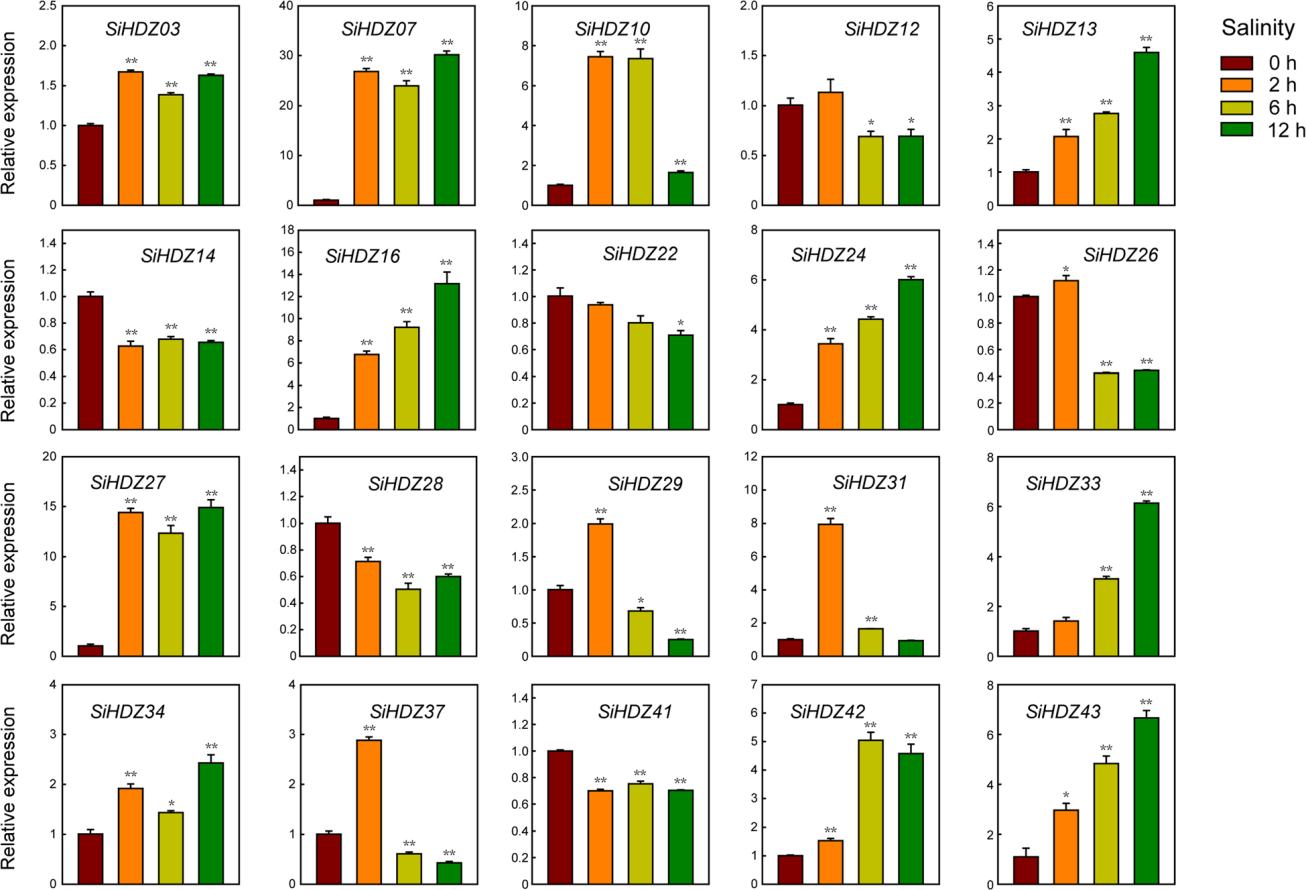
Expression patterns of 20 *SiHDZ* genes under salinity stress. Two-week-old seedlings were subjected to salt (150 mM NaCl) stresses. Relative expression levels of *SiHDZ* genes were analyzed by qRT-PCR, using sesame *SiH3.3* gene as the internal control. Error bars indicate standard deviations (SD) based on three replicates. *P < 0.05; **P < 0.01, *t* test

Numerous evidences showed that HD-Zip genes play a critical role in plant drought and salt tolerance [28, 51–53]. In order to identify candidate *SiHDZ* genes that function in drought and salt stress responses, 20 drought-responsive *SiHDZs* (based on the transcriptome data) from different subfamilies were selected to analyze their expression patterns under osmotic and salinity stresses by qRT-PCR. Overall, the expression levels of all the selected genes were significantly changed in response to osmotic and salinity stresses, but some differences were present among these genes. Under osmotic stress, the expression levels of *SiHDZ03*, *07*, *10*, *13*, *16*, *24*, *27*, *31*, *34*, and *43* were significantly up-regulated through all the time points, while *SiHDZ22*, *29*, *41*, and *42* were induced at certain time points, with the highest expression levels at 6 h, 6 h, 12 h, and 12 h, respectively (Fig. 6 and Additional file 1: Figure S3). However, other *SiHDZ* genes displayed an obvious decrease in expression under osmotic stress at certain time points. Under salinity stress, half of the selected *SiHDZ* genes (*SiHDZ03*, *07*, *10*, *13*, *16*, *24*, *27*, *34*, *42*, and *43*) showed strong up-regulation in expression at all of the time points, while *SiHDZ26*, *28*, *29*, *33*, *37*, and *41* kept low transcription levels in some time points (Fig. 7). In addition, *SiHDZ26*, *29*, *31*, and *37* showed obviously increased expression at 2 h under salinity stress, while the transcripts were dramatically decreased at 6 h and 12 h (Fig. 7). Taken together, we found that the expression of several *SiHDZ* genes, such as *SiHDZ07*, *10*, *27* and *43* was highly induced under both osmotic and salt treatments. These results suggested that these genes might play a vital role in response to multiple abiotic stresses in sesame.

## Discussion

HD-Zip genes encode a family of plant-specific transcription factors involved in various biological processes in plants. In this study, a total of 45 *SiHDZ* genes were identified from the sesame genome (Additional file 2: Table S1). The number of HDZ members in sesame was comparable to that of potato (43) [23], wheat (46) [21], foxtail millet (*Setaria italica*) (47) [54], rice (48) [14, 15], and *Arabidopsis* (48) [6], more than tea plant (33) [22], but less than maize (55) [17] and cassava (57) [20]. These findings suggested that the number of HD-Zip genes is not correlated with genome size of these plant species, which may partly result from tandem duplication and segmental duplication. For instance, over 75% of *Arabidopsis* HD-Zip genes are evolved from segmental duplication and are not clustered on the chromosomes [17, 55]. In cassava, ten HD-Zip genes were identified as segmentally duplicated, while none of the HD-Zip genes were resulted from tandem duplication [20]. In the current study, 36 *SiHDZ* genes were found to be involved in segmental duplication events, whereas no tandem duplicated *SiHDZ* genes were identified, indicating that the segmental duplication was largely responsible for the expansion of HD-Zip gene family in sesame, which is consistent with the results in *Arabidopsis,* cassava and other species [17, 18, 20].

The phylogenetic tree constructed using HDZ proteins from sesame and *Arabidopsis* revealed that the sesame HDZ proteins can be classified into four groups (HD-Zip I-IV) (Fig. 2). In addition, the number of HD-Zip I, II, III, and IV was 16, 10, 9, and 10, respectively (Additional file 2: Table S1; Fig. 2), compared to 17, 10, 5, and 16 in *Arabidopsis* [6], 11, 7, 5, and 8 in grape [16], 17, 18, 5, and 15 in maize [17], and 20, 17, 4, and 5 in wheat [21]. These results revealed that HD-Zip I was the most abundant group, but the number of HD-Zip III proteins in sesame was much higher than those in other plant species, accounting for 20%. Noteworthy, 7 out of 9 *SiHDZ* genes in HD-Zip III subfamily were found to be involved in segmental duplication events (Fig. 2; Additional file 1: Figure S1), suggesting that the HD-Zip III *SiHDZ* genes also mainly expanded during evolution and may play important roles in sesame. Besides, the number of introns in *SiHDZ* family genes varied widely (0–17), but members of the same group have similar exon-intron arrangements (Fig. 3a, b). For example, most *SiHDZ* genes in group III and IV harbored 17 and 8–10 introns, respectively, while members in group I and II had the least number of introns (1–3) (Fig. 3b), which is in accordance with the results in other plant species, such as maize [17], cassava [20], and potato [23]. The differences in the gene structures of different groups suggested group-specific gain or loss of introns that may affect the functional divergence of the different *SiHDZ* group members in sesame. The motif analysis also showed that the distribution of protein motifs in the different groups was noticeably diverse, but the same group had a similar motif construction (Fig. 3a, c), which support their strong evolutionary conservation. These findings suggested that the classification and evolution of the HD-Zip gene family is quite conserved in the sesame, as well as in other plant species.

Increasing evidences have demonstrated that HD-Zip genes participate in various aspects of growth and development in plants [2, 30, 31, 56]. For example, sunflower *HaHB10* (*Helianthus ANNUUS HOMEOBOX 10*) mediates the transition from the vegetative to the flowering stage by activating particular flowering transition genes and plant response to salicylic acid [57]. *AtHB2* regulates red/far-red light effects on the shade avoidance response and specific cell proliferation including lateral root formation [58]. *SiHDZ19* is the homologous gene of *AtHB2* (Fig. 2), and was highly expressed in the root (Fig. 4), suggesting that it may also be involved in root development in sesame. It’s worth noting that some *SiHDZ* genes, especially in HD-Zip I, II, and III, have a broad expression in all tissues (Fig. 4), implying that they participate in the overall development of the sesame. Similar results were also observed in several plant species, such as wheat [21], and potato [23]. However, most HD-Zip IV *SiHDZ* genes displayed distinct tissue-specific expression patterns (Fig. 4), similar to the Group IV genes in cassava [20]. For example, *SiHDZ06*, *11*, *17*, *26*, *28*, and *36* have a much lower expression in roots than in other tissues, while another HD-Zip IV gene, *SiHDZ23*, was specifically expressed in seed (Fig. 4). In cucumber, nearly all HD-Zip IV genes (10 out of 11) showed preferential expression in reproductive organs [59]. Tomato HD-Zip IV genes also showed tissue specific expression patterns, with higher expression in young leaves and flowers [60]. These findings revealed that the functions of different group *SiHDZ* genes were diverged during evolution processes.

Numerous reports have revealed that HD-Zip genes were involved in response to a variety of abiotic stresses in different plant species, such as foxtail millet [54], tea plant [22], wheat [21], and potato [23]. In the present study, the expression levels of over 75% *SiHDZ* genes were found to be affected by drought or salinity stresses based on transcriptome data and qRT-PCR results. Moreover, *SiHDZ03*, *07*, *10*, *13*, *16*, *24*, *27*, *31*, and *43*, have similar expression patterns in response to drought and salinity. A large body of evidence indicates that HD-Zip I proteins are involved in developmental reprogramming in response to environmental stresses [26]. For example, *AtHB7* and *AtHB12*, which are strongly induced by water limiting and ABA, act as mediators that negatively feedback effect on ABA signaling in the plant response to water deficit [26, 27]. The ectopically expression and/or overexpression of the *Arabidopsis AtHB12* or *AtHB7* gene, or their homologs in *M. truncatula* (*MtHB1*), sunflower (*HaHB4*), and maize (*ZmHDZ4*), enhanced tolerance to drought or salinity stress in transgenic plants [29, 61–63]. Some of the HD-Zip I genes in sesame, such as *SiHDZ07*, *10*, *16*, *24*, *27*, *31*, *37*, and *43*, were significantly induced in response to drought or salt stress. Notably, the expression of *SiHDZ07*, *16*, and *31*, homologs of *Arabidopsis AtHB12* and *AtHB7* genes, was significantly up-regulated by both drought and salinity stresses, indicating that these genes may regulate drought and salt tolerance through an ABA-dependent pathway. In addition, *SiHDZ43*, like its homologs *AtHB21* and *AtHB40*, was up-regulated under drought and salinity stress. Three HD-Zip II members, *SiHDZ03*, *13*, and *42*, were also significantly up-regulated under drought and/or salt treatment, thereby suggesting their possible role in abiotic stress responses. These results suggest HD-Zip I and II genes may play a vital role in regulating plant tolerance to the adverse environmental conditions.

## Conclusions

In this study, we identified 45 HD-Zip genes from sesame at the whole genome level. Phylogenetic analysis identified four subfamilies (HD-Zip I-IV) in the HD-Zip gene family, which was further supported by the analysis of their conserved motifs and gene structures. Transcriptomic analysis revealed some constitutively or tissue-specific expressed HD-Zip genes. Expression profiles of *SiHDZ* genes under various abiotic stress treatments indicated that over 75% *SiHDZ* genes are involved in abiotic stress signaling, and members of HD-Zip I and II subfamilies may play a vital role in regulating plant response to abiotic stresses. Together, these data provide useful information for functional characterization of *SiHDZ* genes and extend our knowledge of abiotic stress response in sesame.

## Methods

### Genome-wide identification of HD-zip family genes in sesame

To identify HD-Zip protein in sesame, all proteins sequences were downloaded from the *Sesamum indicum* genome database (Sinbase, http://ocri-genomics.org/Sinbase/index.html) [64]. A local protein database was constructed and searched against known HD-Zip protein sequences collected from *Arabidopsis* using a local protein basic local alignment search (BLASTP) program with an E-value cut-off < 10–5 and an identity of 50% as the threshold. HMM profile of the homeodomain (PF00046) and the leucine zipper domain (PF02183) were download from the PFAM database and used for local Hidden Markov Model (HMM) search by HMMER3.0 [65]. Subsequently, all obtained protein sequences were further examined by SMART (http://smart.embl-heidelberg.de/) to confirm the presence of the HD and LZ domains. Finally, a total of 45 non-redundant sesame HD-Zip encoding genes were identified.

### Phylogenetic analysis and gene duplication

The protein sequences of HD-Zip from sesame and were *Arabidopsis* used to construct the phylogenetic tree by MEGA 5.2, using the neighbor-joining (NJ) method with 1000 bootstrap replications [66]. Gene duplication was analyzed with MCScanX following the method described previously [67].

### Gene structure and protein conserved motifs analysis

The exon/intron organization of the HD-Zip genes in sesame was performed with Gene Structure Display Server (GSDS) (http://gsds.cbi.pku.edu.cn/index.php). Conserved motifs present in SiHDZs were identified using MEME (Multiple Em for Motif Elicitation) v4.11.4 (http://meme-suite.org/tools/meme).

### Expression profiling of SiHDZ genes using available transcriptome data

To gain insight into the tissue-specific gene expression patterns of HD-Zip gene, transcriptome data for six tissues (root, stem, flower, leaf, capsule and seed) were extracted from Sesame Functional Genomics Database (SesameFG, http://www.ncgr.ac.cn/SesameFG) [49]. To analysis the expression profiles of *SiHDZ* genes in response to drought stress, the transcriptome data (accession number SAMN06130606) were used [50]. The hierarchical cluster analyses of gene expression were performed using Cluster 3.0 software [68], and heatmaps were visualized with TreeView [69].

### Plant materials and treatments

In this study, Two-week old seedlings of Sesame cultivar Zhongzhi No. 13 were used to examine the expression patterns of *SiHDZ* genes under osmotic and salt stresses. Plants were grown in a growth chamber at 28 ± 2 °C with a 16-h light/8-h dark photoperiod, and exposed to different stresses as described previously [70]. The shoots tissues were collected at 0, 2, 6 and 12 h after treatment. Three biological replications were conducted per sample.

### Quantitative real-time RT-PCR

Total RNA isolation and the first-strand cDNA synthetization were performed as described previously [70]. The gene-specific primers used in this study are listed in Additional file 3: Table S2. Quantitative real-time RT-PCR (qRT-PCR) was performed according to the protocol described previously [70]. Significant up- and down-regulated genes were determined as *p* < 0.05 or *p* < 0.01 using *t* test.

## Supplementary information

**Additional file 1:** **Fig. S1.** Segmental duplicated *SiHDZ* genes on 16 linkage groups. Red lines indicate duplicated *SiHDZ* gene pairs. Grey lines indicate collinear blocks in whole sesame genome. **Fig. S2.** The logos of 20 conserved motifs in SiHDZ proteins. **Fig. S3.** Expression profiles of stress marker genes under osmotic and salinity stress treatments.

**Additional file 2. Table S1.** HD-ZIP transcription factor gene family in sesame.

**Additional file 3. Table S2.** List of primers used for quantitative real-time RT-PCR analysis.

## Data Availability

Gene sequence information of HD-Zip in sesame is available at the *Sesamum indicum* genome database (Sinbase, http://ocri-genomics.org/Sinbase/index.html).

## Abbreviations

AtHB: *Arabidopsis thaliana* homeobox
HD: Homeodomain
HD-Zip: Homeodomain-leucine zipper
HMM: Hidden Markov model
qRT-PCR: Quantitative real-time reverse transcription-polymerase chain reaction
SiHDZ: *Sesamum indicum* homeodomain-leucine zipper
TF: Transcription factor
