# Supplementary material for: Genome-wide characterization and expression analysis of the HD-Zip gene family in response to drought and salinity stresses in sesame
Source: BMC Genomics. 2019 Oct 16;20:748. doi: 10.1186/s12864-019-6091-5 (PMC6796446; doi:10.1186/s12864-019-6091-5)
Supplement: Supplementary file 1 — Additional file 1: Fig. S1. Segmental duplicated SiHDZ genes on 16 linkage groups. Red lines indicate duplicated SiHDZ gene pairs. Grey lines indicate collinear blocks in whole sesame genome. Fig. S2. The logos of 20 conserved motifs in SiHDZ proteins. Fig. S3. Expression profiles of stress marker genes under osmotic and salinity stress treatments. [file 12864_2019_6091_MOESM1_ESM.docx]

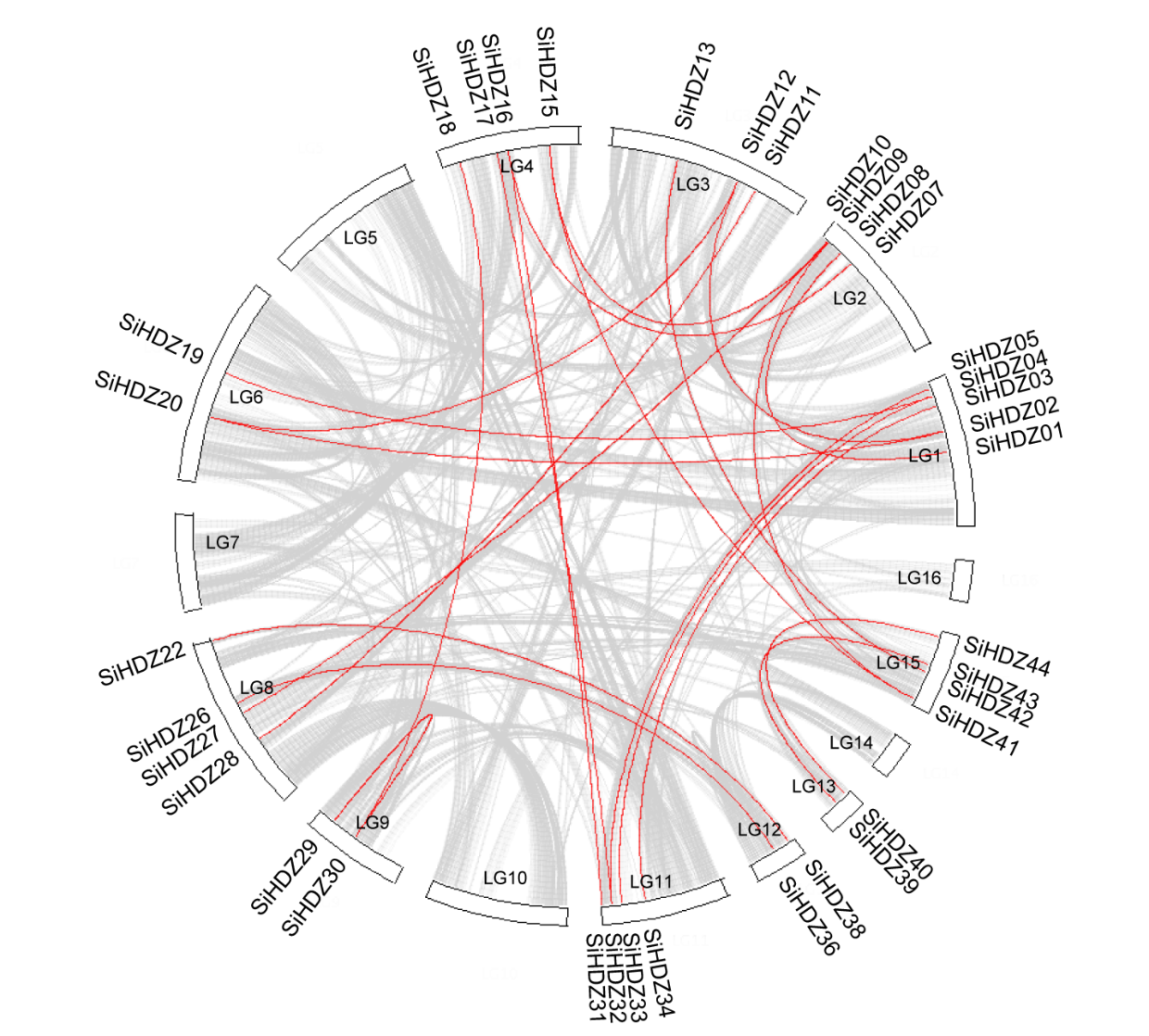


**Figure S1. Segmental duplicated *SiHDZ* genes on 16 linkage groups**

Red lines indicate duplicated *SiHDZ* gene pairs. Grey lines indicate collinear blocks in whole sesame genome.


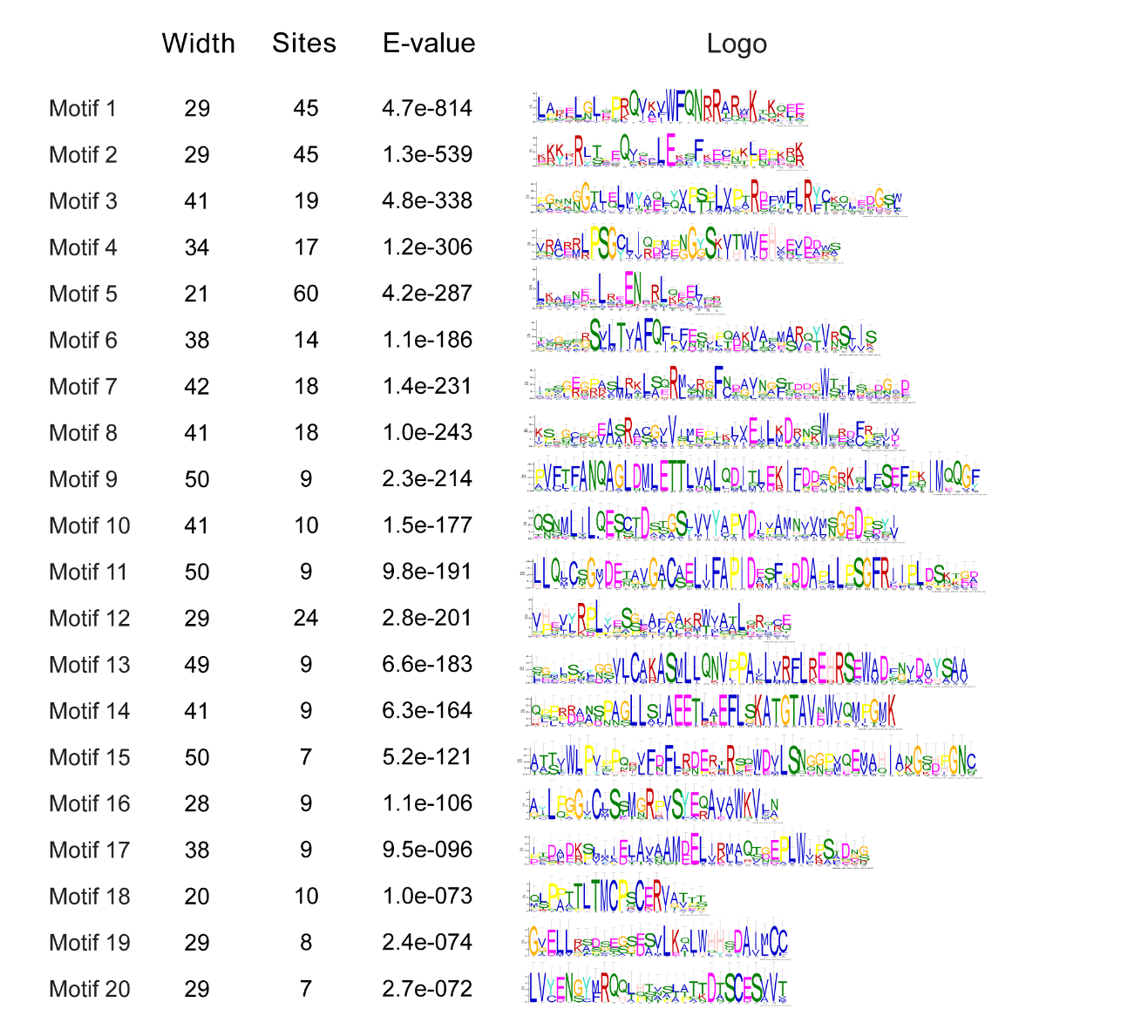


**Figure S2. The logos of 20 conserved motifs in SiHDZ proteins.**

**
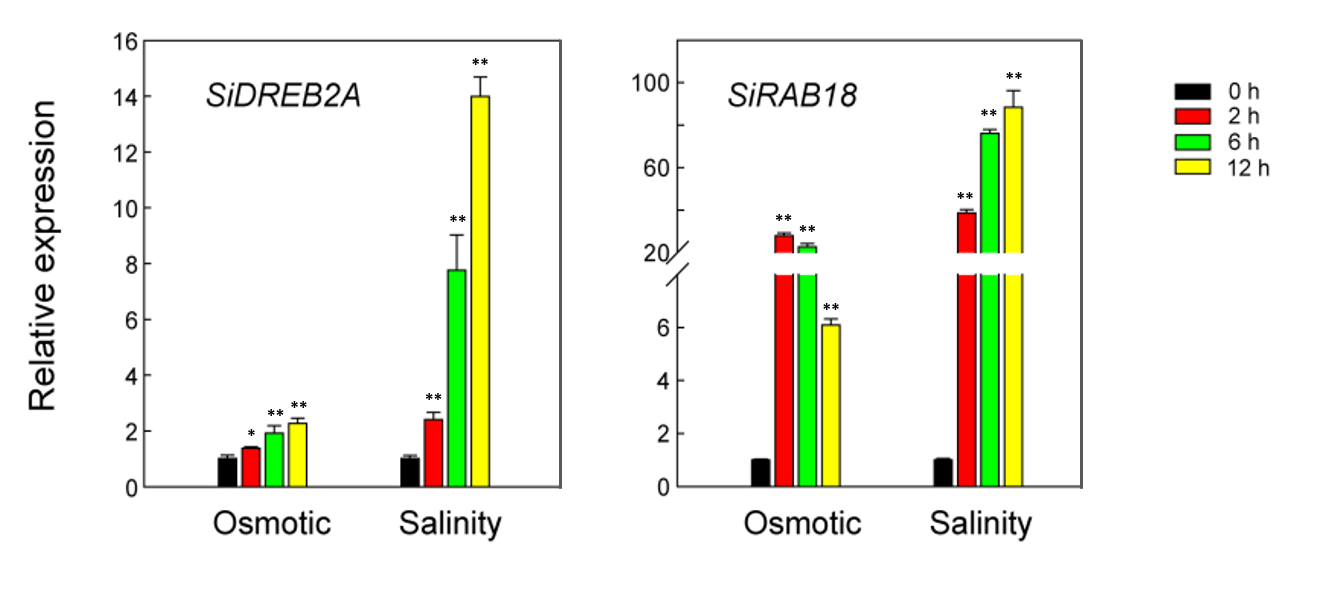
**

**Figure S3. Expression profiles of stress marker genes under osmotic and salinity stress treatments.** Two-week-old seedlings were subjected to osmotic (15 % PEG 6000) and salt (150 mM NaCl) stresses. Relative expression levels of known stress-inducible genes, *SiDREB2A* and *SiRAB18*, were analyzed by qRT-PCR, using sesame *SiH3.3* gene as the internal control. Error bars indicate standard deviations (SD) based on three replicates. **P* < 0.05; ***P* < 0.01, *t* test.
